# Assessment of the estuarine shoreline microplastics and mesoplastics of the River Itchen, Southampton (UK) for contaminants and for their interaction with invertebrate fauna

**DOI:** 10.1007/s11356-023-31396-6

**Published:** 2023-12-27

**Authors:** Deanna L. G. Rose, Malcolm D. Hudson, Sargent Bray, Pawel Gaca

**Affiliations:** 1 School of Geography and Environmental Science, University of Southampton, Highfield, Southampton, SO17 1BK UK; 2 National Oceanography Centre, University of Southampton, European Way, Southampton, SO14 3ZH UK

**Keywords:** Plastic debris, Persistent organic pollutants, Marine protected areas, Trace metals, Amphipods, Ecological impacts, Southampton Water, The Solent

## Abstract

The presence of shoreline microplastics (1–5 mm) and mesoplastics (5–25 mm) in estuarine ecosystems is ubiquitous, but there remains little data on their composition, contamination status and ecological impacts. Chessel Bay Nature Reserve, situated in the internationally protected Itchen Estuary in Southampton, UK, has serious issues with shoreline plastic accumulation. In evaluating potentially adverse ecological impacts, the influence of quantities of shoreline microplastic (mp) and mesoplastic (MeP) material and adsorbed contaminants (PAHs and trace metals) on the biometrics and population dynamics of the burrowing supralittoral amphipod, *Orchestia gammarellus*, was assessed in this study. mp/MeP concentrations were variable in surface (0–42%: 0–422,640 mg/kg dry sediment) and subsurface horizons (0.001–10%: 11—97,797 mg/kg dry sediment). Secondary microplastics accounted for 77% of the total microplastic load (dominated by fragments and foams), but also comprised 23% nurdles/pellets (primary microplastics). Sorption mechanisms between contaminants and natural sediments were proposed to be the main contributor to the retention of PAHs and trace metal contaminants and less so, by mp/MeP. *O. gammarellus* populations showed a positive correlation with microplastic concentrations (Spearman correlation, *R* = 0.665, *p* = 0.036). Some reported toxicological thresholds were exceeded in sediments, but no impacts related to chemical contaminant concentrations were demonstrated. This study highlights a protected site with the severe plastic contamination, and the difficulty in demonstrating in situ ecotoxicological impacts.

## Introduction

Microplastic pollution in the marine environment is not only a pervasive global environmental challenge but reflects a subset of the larger plastic pollution issue arising from mismanagement across the value chain from producer to consumer (Geyer et al. 2017). As many common polymers are lightweight materials, they are easily dispersed by the wind and float along water courses, ending up along coastlines and in estuarine, pelagic and benthic regions of the marine environment (Browne et al. 2008). Whether entering as primary microplastics in the form of pre-production pellets or nurdles, or as fragments, fibres or foams of weathered or degraded plastic material, as secondary microplastics (Cole et al. 2011), their highly durable nature has translated into their long-term persistence and furthermore may threaten organisms across all trophic levels of the marine energy hierarchy (Andrady 2011). Microplastics, which are defined as regularly or irregularly shaped synthetic polymer particles with dimensions between 1 μm and 5 mm (Frias and Nash 2019), have been found to be ingested during normal feeding mechanisms (whether accidentally or selectively) by macroinvertebrates (Cole et al. 2013), bivalves (Van Cauwenberghe and Janssen 2014), finfish (Lusher et al. 2013; Arias et al. 2019), turtles (Caron et al. 2018) and seabirds (Weitzel et al. 2021). This has led to incidences of gut blockage and false satiety (Santos et al. 2020; Hierl et al. 2021), and the exposure to chemical compounds adsorbed to the surface of microplastics, given their hydrophobic nature (Bakir et al. 2016). Mesoplastics (5–25 mm, Shim et al. 2018) are important pollutant intermediaries propagated via weathering mechanisms of larger plastic items (macroplastics, > 25 mm), and represent point sources of secondary microplastics. The latter has been recorded in this manuscript (> 5.6 mm), however, maintaining a stronger focus on microplastics (< 5.6 mm).

Given optimal conditions such as temperature, pH and availability of complementary functional groups, trace metals, polycyclic aromatic hydrocarbons (PAHs), polychlorinated biphenyls (PCBs), along with plasticizers such as phthalates, including bisphenol A (which are often also present as additives to plastics), have been found as adsorbates of microplastics recovered from marine habitats (Bakir et al. 2014; Brennecke et al. 2016; Liu et al. 2021)**.** PAHs and trace metals represent substances that exist naturally but have become enriched due to anthropogenic influences (Wolska et al. 2012). Both can elicit sub-lethal and lethal effects. Persistent organics such as PAHs have no biological function but can be toxic or carcinogenic (Honda and Suzuki 2020), while trace metals, which can be essential or non-essential to organisms’ physiology, may be detrimental at high concentrations (Huang et al. 2020). Exposure of marine organisms to these compounds has, often, been met with the complexity of attributing a consequence of physiological dysfunction (Fisner et al. 2013) and the dynamics between the microplastic particles and natural materials are not well understood (Hee Joo et al. 2021). Rochman et al. (2013), however, cautiously demonstrated heightened hepatic stress in the fish exposed to plastic nurdles contaminated with adsorbed organic contaminants versus uncontaminated nurdles. Despite the difficulty in clearly demonstrating toxicological effects of microplastics in environmental situations, there is no doubt that accumulation of waste material in the environment is undesirable and can be expected to lead to increasing adverse effects over time (Lusher 2015).

Marine sediments represent a hotspot for microplastics, where they either sink to benthic sediments due to biofouling or wash ashore by wave and tidal action (Browne et al. 2011). Estuarine habitats have had increased scientific attention in this respect, due to their vulnerability as intermediary systems between river systems and the open ocean (Stead et al. 2020; Xu et al. 2020). Given their location and characteristics, estuaries can filter sedimentary material flowing from upstream sources and ultimately become microplastic accumulation zones, especially in proximity to regions of high population density (Díaz-Jaramillo et al. 2021). Thus, microplastics amass within the shoreline sediments as physical and chemical contaminant complexes and are potentially bioavailable to indwelling estuarine biota (Hodgson et al. 2018).

As a measure of ecological impact in estuaries, shoreline gammarid amphipods often reflect environmental conditions, due to their sensitivity to physical and chemical changes to their environment. They have been used as bioindicators of trace metal and persistent organic pollutants, and human trampling impacts on beaches (Ugolini et al. 2008; Ungherese et al. 2010). Key sedimentation processes are enabled by the foraging mechanisms of these amphipods, which dwell within burrows of the sediment of the intertidal zone and consume the detritus of algal debris and plant material. This has the twofold benefit of nutrient cycling, as many amphipods are important food sources at the base of shoreline food chains (Ianilli et al. 2018). Their essentiality may, however, be threatened as impacts of microplastics on gammarids have been demonstrated in past research. Tosetto et al. (2016) demonstrated that microplastic ingestion by the sandhopper *Platorchestia smithi* resulted in impaired locomotion and a retardation in predator and desiccation avoidance. Furthermore, ingestion of microplastics can potentially increase the exposure of these amphipods to toxic adsorbed persistent contaminants, (see above), which could impair physiological function (Scopetani et al. 2018). Though previous studies conducted in Italian contexts have demonstrated the potential of microplastic ingestion in natural and laboratory-regulated conditions (Ugolini et al. 2013; Ianilli et al. 2018), incidences of this nature are yet to be identified within the UK or elsewhere.

In the UK, estimates of annual plastic waste input into the marine environment vary greatly between 10,000 and 17,000 tonnes (Thompson 2017), and impact a significant portion of the approximately 530,000 ha estuarine resource (Davidson 2018). Though 44% of UK estuaries have national and international conservation designations, an extensive citizen science programme over a 25-year period revealed high densities of marine litter in marine-protected areas of England’s southeast and southwest (Nelms et al. 2020). The sediments across many of these regions were also confirmed as microplastic pollution hotspots by Green and Johnson (2020), highlighting that these designations lack effective conservation objectives that safeguard these ecosystems against plastic and microplastic pollution. Furthermore, there exists a deficit on data regarding the ecological impacts of microplastics on features of marine protected areas in the UK (Defra n.d.). The Solent in southern England is one such heavily protected location with numerous designated sites, and has a long history of trace metal, petrochemical and microplastic accumulation (Croudace and Cundy 1995; Gallagher et al. 2016), with recent local concern regarding accumulation of nurdles and other debris within Chessel Bay Nature Reserve, situated in the nationally and internationally protected Itchen Estuary.

This study seeks to investigate the abundance, nature of and contamination status of shoreline microplastics amongst natural sediments at the Chessel Bay, and aims to make a preliminary evaluation of the impact on populations of the dominant macroinvertebrates (amphipods) present.

## Methodology

### Study area

**Fig. 1:**
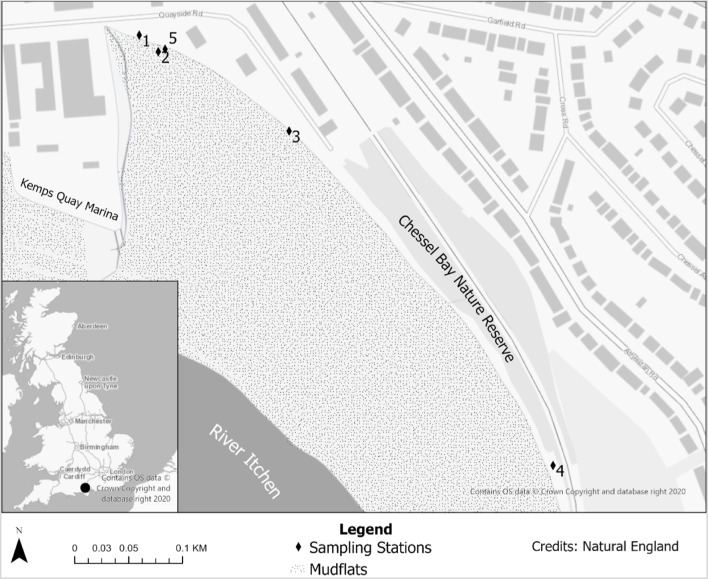
Locations of sampling stations within the study area situated along the shoreline of Chessel Bay Nature Reserve in Southampton, England

The study took place within Chessel Bay Nature Reserve in Southampton, Hampshire, Southern England (50.9154° N, 1.3746° W) (Fig. 1)—a 12.9-ha undeveloped shoreline within the Southampton conurbation comprising mainly of mudflats, saltmarsh and a narrow strip of woodland. Chessel Bay sits on the eastern bank of the River Itchen, one of three tributaries within the Solent-Southampton Water estuarine complex, which currently holds nature conservation designations under the Ramsar Convention for Wetlands, Natura 2000 Habitats Directive (Special Protected Area and Special Area of Conservation) and the UK Wildlife and Countryside Act (1981) (Site of Special Scientific Interest) (Chessel Bay 2021). Much of the sediment and pollutant deposition is influenced by the precipitation and surface run-off, residential, commercial and industrial activities adjacent to and upstream of the site (Raymont 1972).

During the preliminary site visit (June 2021), important pollution characteristics and ecological dynamics of the intertidal zone were noted: (i) shoreline sediments were inundated by significant quantities of nurdles, along with fragments of larger weathered plastic debris; (ii) algal mats on the water’s surface and along the shoreline indicated eutrophication; and (iii) halophyte vegetation distribution and abundance was quite variable: sea club rush (*Scirpus maritimus*), common reed (*Phragmites australis*), orache (*Atriplex hastata*), sea couch grass (*Agropyron pungens*), sea aster (*Aster tripolium*) and mud rush (*Juncus gerardii*) distributed patchily within the sample area in the upper shore. Five sampling stations along the upper shoreline were purposefully targeted: Three of these stations are considered here as “hotspots” for plastic (microplastic and mesoplastic) (stations 1, 3 and 4), as well as a station for comparison which was obviously less inundated by plastics (station 2). A further location was demarcated as a “clean” or “control” station (station 5), where presence of microplastics, mesoplastics and other anthropogenic debris was much less evident—although some plastics were present all over the site. Such visual impressions have been the basis of misleading media interpretations of these incidences (Henderson and Green 2020). This study will, therefore, test the visual assumptions of the perceived pollution with empirical evidence.

### Sediment and amphipod sampling

Sampling of the sediments for microplastics and amphipod specimens took place in July 2021 during neap tides from the upper shore at the highest strandline. The depths of the vertical sediment horizon at each station were inconsistent across the study area, and therefore, the distinctions between the organic layer (humus) [representing the surface layer], mineral-humus layer [representing the sub-surface layer] and the partly weathered rock (gravel and shingle) layer were measured to classify these layers. The surface layers averaged 0–2.4 cm and the subsurface layers, 2.4–6 cm (see Table 3 for description of each station and horizon depths). The sediment was sampled for microplastics based on modifications of the protocol by Wessel et al. (2016) and Hanke et al. (2013). At each of the five sampling stations (Fig. 1), large pieces of wood, leaves and other natural pieces of debris were removed from the surface of the intertidal sediments. Using a hand trowel, duplicate samples of the surface and sub-surface layers were sampled from a 0.25 m × 0.25 m area delineated by a quadrat and placed in pre-labelled and pre-cleaned sampling jars and stored in a cooler with ice packs to be brought to the laboratory for fractionation and other quantitative analysis. A total of 20 samples (10 surface and 10 subsurface) were collected, with 2 samples of the surface and 2 samples of the sub-surface collected at each of the 5 stations. The location of each sampling station was recorded with a Garmin etrex10 Handheld GPS unit. Average moisture content of the surface sediment was obtained from five readings taken at each station using a HH2 Delta-T moisture probe (Table 3).

Amphipods were passively sampled using the pitfall trap method (Pavesi et al. 2009) for which slender plastic jars (diameter 6 cm; height 12 cm) were buried at neap tide (to avoid inundation) in each station, with the opening level with the surface of the sediment. The traps were retrieved 24 h later and collected specimens transferred to labelled sample jars containing fresh 70% ethanol, which quickly euthanised and fixed the amphipods’ biology for further analysis.

### Microplastic characterisation

#### Visual sorting and classification

The bulk sediment samples were thoroughly mixed and sub-sample masses (50 g and 100 g) weighed. Due to constraints of the project timeline and the extraordinary COVID-19 restrictions on laboratory accessibility at the time, smaller sub-sample masses were used during the progression of the laboratory work (8 sample duplicates weighed 100 g initially, but 50 g sub-samples were taken from the remaining 12 samples). These were separated through a sieve cascade for further sorting and classification following: 5.6 mm, 2 mm and 1 mm (Crawford and Quinn 2017). Due to the difficulty of accurately qualifying plastic material with the naked eye below 1 mm, the size classes maintained for the study were based on this sieve cascade: > 5.6 mm, 2–5.6 mm and 1–2 mm. This approach deviates from the accepted size classes for microplastics (see above), but the > 5.6-mm portion, though mesoplastics rather than microplastics, by definition (5–25 mm; GESAMP 2019) was essential to the study as each item represents a potential point source of secondary microplastics in the study area. Masses were moisture-corrected based on percentage moisture readings obtained in situ from each sampling station. Results thus were normalised based on mass concentration as mg mp/MeP per kg dry sediment. While this is a typical approach of determining moisture content of sediments in the field, there is some error in this estimation (Yang and Davidson-Arnott 2005). Nevertheless, it is preferable to assess plastic content per estimated dry weight, given the variability in moisture content recorded (Table 3). The oven-drying approach in microplastic studies has been cautioned over 60 °C (Munno et al. 2018) as high temperatures disfigure some common polymers that may soften or undergo glass transition (McKeen 2014), which would hinder effective sorting and characterisation and assessment for contaminants. Reported dry weight protocols, exceeding this threshold, such as Peng et al. (2017) [70 °C for 24 h], Masura et al. (2015) [90 °C overnight] and Heiri et al. (2001) [ca. 105 °C for 12–24 h] were not suitable.

With the aid of the Nikon SMZ1000/C-W10xB/22 light microscope with gooseneck lighting, each fraction was enumerated and categorised by morphology (fragment, fibre/filament, pellet, foam, film) and colour (black, blue, brown, cream, green, white, opaque, orange, pink, purple, red, transparent, yellow) according to the standardised Size and Colour System (SCS) (Crawford and Quinn 2017).’Pellet’ in this context refers to pre-production nurdles, but also to ‘biobeads’ used in wastewater treatment infrastructure, which have similar morphology and polymer compositions. It would have been preferable to use spectroscopic methods (e.g. FTIR (Fourier transfer infrared)) for characterisation of the particles, but this was unavailable during the study—in part due to Covid-19 restrictions. The characterisation approaches employed in the study have been widely applied and cited in recently published work, e.g., Blair et al. (2019), Irfan et al. (2020) and Mbedzi et al. (2020). The lower size limit of 1 mm avoids the challenges of visual identification at smaller sizes discussed by Loder and Gerdts (2015). Misidentification of microplastics in the samples was avoided with guidance from Nor and Obbard (2014): no visible cellular or organic structures; fibres equally thick and non-tapered across the entire length; homogenous colour; fibres are not segmented or as twisted flat ribbons; and particles are not shiny. A final weight of the plastic portion of each fraction was taken using an analytical balance. A percentage account of microplastics in the sediment samples was later determined.

### Analysis of organics and trace metals

#### Polycyclic aromatic hydrocarbons

A portion of homogenized whole sediment/micro(meso)plastic samples were transferred to labelled petri dishes and freeze-dried overnight. These whole samples comprised of soil, mixed woody debris and other organic material and nurdles. The freeze-dried portion was mixed and transferred to labelled stainless steel extraction cells, with the weight taken of the material fitted into the cell. The cells were loaded on the carousel of the Dionex ASE 350 Accelerated Solvent Extractor and extracted with 9:1 *n*-hexane/dichloromethane. The collected extracts were then analysed on a Thermo Trace 1310 gas chromatograph coupled to a Thermo TSQ8000 mass spectrometer operating in single quadrupole mode. The resulting spectrum was post-processed to evaluate the presence of four common PAH compounds: phenanthrene, fluoranthene, benz[a] anthracene and benzo [k] fluoranthene, as synthetic descriptors of the composite sample (containing natural and plastic material as mentioned before).

**Fig. 2:**
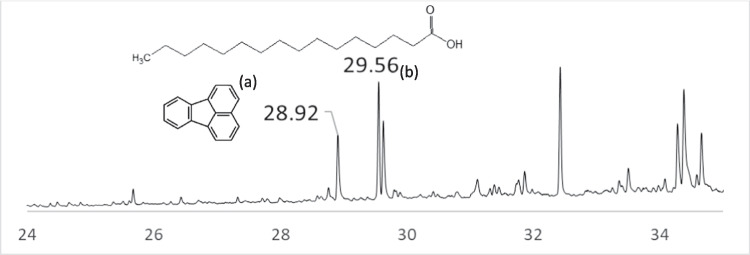
Example PAH spectrum with highlighted peaks: **a** fluoranthene and **b** palmitic (hexadecanoic) acid for the determination of the FA/PAH ratio

PAH diagnostic ratios are commonly reported in literature as forensic indicators of their origins and ecotoxicological relevance in marine sediments (Botsou and Hatzianestis 2012; Han et al. 2019). Typically, pyrogenic sources are attributed to the combustion of fossil fuels, and petrogenic sources, though less common, are related to the incomplete combustion of organic matter (Fisner et al. 2013). This approach was modified to describe the relative signal of synthetic organic compounds to natural organic compounds in the form of fatty acids. A sedimentary fatty acid (FA) profile reflects the specificity to which they originate from organic matter and are commonly employed as molecular biomarkers (Gardade et al. 2021). For this purpose, palmitic acid, related to the origin of detrital materials (Gardade et al. 2021) and the most abundant saturated fatty acid in organisms (Rhead et al. 1971), reflected the FA term in the ratio: FA/PAH. The area under the curve of each peak selected (Fig. 2) was used to compute this ratio.

#### Trace metals

Trace metal concentrations were evaluated with modifications to Holmes et al. (2012): 30 microplastic particles of varying morphologies were selected randomly from samples at the three stations selected as microplastic pollution hotspots. Samples were weighed into 50-mL plastic vials previously pre-cleaned by soaking in 10% (v/v) nitric acid solution, agitated for 48 h in approx. 10 mL of de-ionised water (> 18 MΩ) to remove any organic matter and sediment sticking to the surfaces of the microplastics. The microplastic particles were removed from this wash solution and transferred to new pre-cleaned and pre-weighed 50-mL plastic vials. They were subsequently dried at 60 °C for a period of 1 week to determine the dry mass of the microplastic particles before metal extraction in 10 mL of 20% aqua regia (1 HNO_3_/3 HCl; spectral analysis purity grade). The samples were then agitated on a rotary shaker for 48 h. The resulting extracts with de-ionised water washings were then transferred to clean plastic vials and evaporated to dryness on medium heat (ca. 70 °C) before re-dissolving the residue in 3% (v/v) nitric acid with In and Re internal spike for ICP-MS analysis.

Initial water wash solutions with the removed sediment/organic matter were treated using the same procedure that was applied to the microplastic samples, resulting in two separate sample solutions for each collected sample. Both solutions were analysed using Agilent 8800 Triple Quadrupole ICP-MS. The instrument was run using a collision chamber with He gas to reduce the polyatomic interferences to the analysed metal suite: Al, Cd, Co, Cr, Cu, Ni, Pb and Zn (essential and non-essential elements that exhibit lethal consequences to biota at high concentrations in their bodies). The instrument was calibrated using multi-elemental calibration standards, traceable to NIST. A set of three blank samples were analysed alongside the microplastic samples to allow for a suitable correction of the obtained results.

### Amphipod classification and dissection

*Orchestia gammarellus* were the only amphipod species in the pitfall samples, based on the species description by Lincoln (1979), and were differentiated based on sex, length and reproductive status (with/without eggs or hatched juveniles) according to Moore and Francis (1986). Males were distinguished by their claw on gnathopod 2; females, with large overlapping or setose oostegites, categorised as ovigerous, carrying eggs, with hatched young in their brood pouch, or none; and juveniles, individuals with unidentifiable sexual characteristics and a total body length of less than 8 mm. During the biometric evaluation of the amphipods, one ovigerous female appeared to contain a microplastic fragment within the brood pouch, but this moved during the handling to capture a photograph (Fig. 9), and we were unable to examine it further. No other incidences of this nature occurred in the other amphipods inspected.

### Statistical analysis

Non-parametric statistical tests were employed on all data variables, i.e., microplastic concentrations, moisture content and amphipod population, due to non-normality of microplastic concentrations, according to a Shapiro–Wilk normality test. The Kruskal–Wallis test was performed to evaluate the degree to which differences in trace metal concentrations and relative humidity between stations varied. In instances of significant differences, Dunn’s post hoc test (with Bonferroni correction) was conducted to establish which variables were different. Mann Whitney *U* test was used to determine significant differences of microplastic sediment concentrations between the sub-surface and surface horizons. The metric of a linear model was constructed to describe the influence of surface sediment microplastic concentrations on that of the sub-surface sediment. Statistical significance was not determined across stations and horizons for the FA/PAH diagnostic ratio since it was a semi-quantitative metric informing the study of the relative dominance of pyrogenic and pyrolytic PAHs to that of the organic signature in the Chessel Bay shoreline sediments. Furthermore, concentrations of secondary contaminants (PAHs and metals) and microplastics were assessed for a significant effect on amphipod population dynamics and biometrics (length) across the study area by the use of Spearman’s rank correlation in the web-based Wessa (2022) statistics software (supported by R). All other analyses were carried out at a 95% confidence level in Microsoft Excel with XLSTAT 2021 (V. 3.1.1183).

### Contamination control

Quality control measures were employed at each stage of the study to minimise the contamination of samples with airborne microplastics, or cross-contamination. Briefly, all sampling equipment was thoroughly pre-cleaned with distilled water: stainless steel hand trowels were used and samples stored in brand-new plastic containers which were carefully handled to prevent breakage and contaminate the samples. A cotton laboratory coat was always worn in the laboratory during sample preparation and analysis. All glassware, sieves and stainless steel spatulas were properly precleaned with mild detergent and distilled water before use. Samples were always covered with watch classes when not in use. Laboratory windows were sealed during the time of analysis and only central air conditioning served the ventilation needs of the room. This minimised the introduction of airborne microplastics into the laboratory environment.

Laboratory blanks containing only deionised water were passed through the sieve cascade (5.6 mm, 2.5 mm, 1 mm) and then through the entire analytical process to monitor potential contamination on each day of characterisation. These were beakers of deionised water that were left exposed to the ambient laboratory air, to identify potential airborne contamination. Microscope examination of the filtrate found no visible airborne particulates in the blanks analysed.

## Results

### Sediment microplastics: concentrations and characteristics

#### Microplastic concentrations in the sediment

**Fig. 3:**
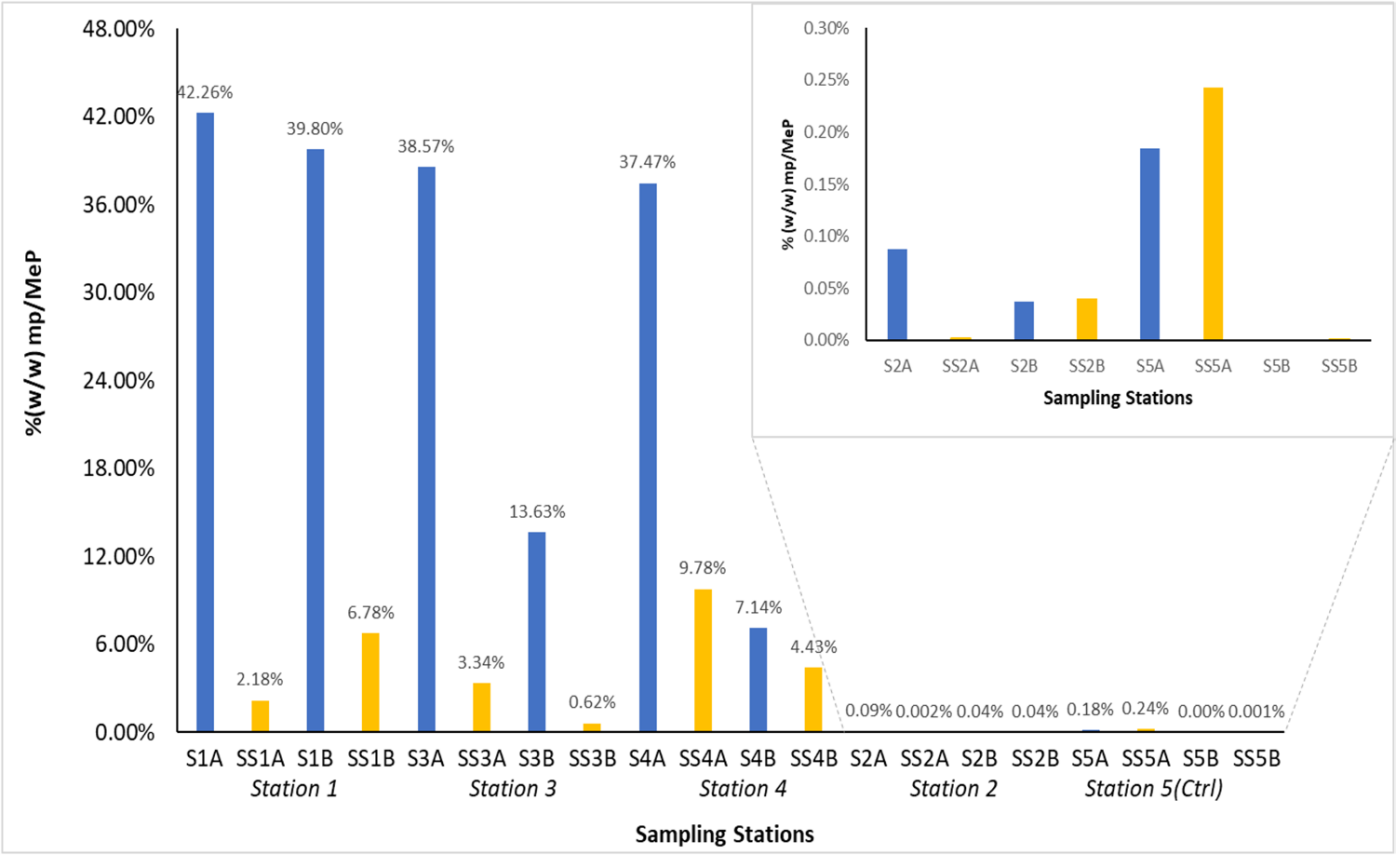
Mass fraction (%w/w) of mp/MePs at the sampling stations across the Chessel Bay intertidal sediments. **Blue** bars represent the surface horizons, and the **gold** bars represent the subsurface horizons. Duplicates were analysed of both surface and sub-surface layers of the sediment. S1A and S1B indicate the duplicates of the surface sediment sample at station 1; SS1A and SS1B indicate the duplicates of the sub-surface sediment sample at station 1. This coding is also applicable to the other stations. Stations are organised along the *x*-axis in the order of most polluted to least

Microplastic and mesoplastic contamination was evident across all sampling stations on the shore, and concentrations were significantly different between stations (Kruskal–Wallis, *H*(4) = 13.943, *p* < 0.001). Moreover, within just a 0.25 m × 0.25 m area of the sediment horizon of the stations along the Chessel Bay supralittoral, microplastic concentration was variable both in the surface and sub-surface layers and in their duplicates from the same locations. Surface-level mp/MeP concentrations ranged from 372 to 422,640 mg/kg sediment (0.003–42.3%) corrected for moisture content, with station 1 exhibiting the greatest contamination, where microplastics and mesoplastics accounted for up to 42.3% of the sediments in the surface horizon (Fig. 3). The sub-surface concentration was almost two-100-fold less of the microplastic load, containing 2184 mg mp/MeP per kg (0.2%).

Across the study area, this trend was described by the linear model mp_sub-surface_ = 0.1236mp_surface_ + 0.0053 (*R*^2^ = 0.49), which predicts the sub-surface microplastic concentrations based on surface contamination load with a degree of 49% accuracy. Despite smaller quantities of mp/MeP in the sub-surface than the surface, a Mann–Whitney *U* test indicated no significant differences in concentration across the horizons (*U* = 33, *p* = 0.218). At station 3, surface sediments between duplicates contained 385,684 mg mp/MeP per kg dry sediment (39%) and 136,297 mg mp/MeP per kg dry sediment (14%) respectively. Similarly, at station 4, surface microplastic concentrations of the two duplicates were 374,707 mg mp/MeP per kg dry sediment (37%) and 71,355 mg/kg dry sediment (7%). The visibly least polluted area (station 2) was, as expected, much lower in mp/MeP with nearly 2 to 4 orders of magnitude less than the three pollution hotspots (372–876 mg/kg dry sediment at the surface 0.04–0.09%; 16.72–397.08 mg mp/MeP per kg dry sediment 0.002–0.04). Moisture levels across stations were not significantly different (Kruskal–Wallis, *H*(4) = 4, *p* = 0.406), although stations 2 and 5 (as less polluted stations) had greater retention of moisture than stations 1, 3 and 4 (Table 3).

#### Microplastic morphologies and colours

**Fig. 4:**
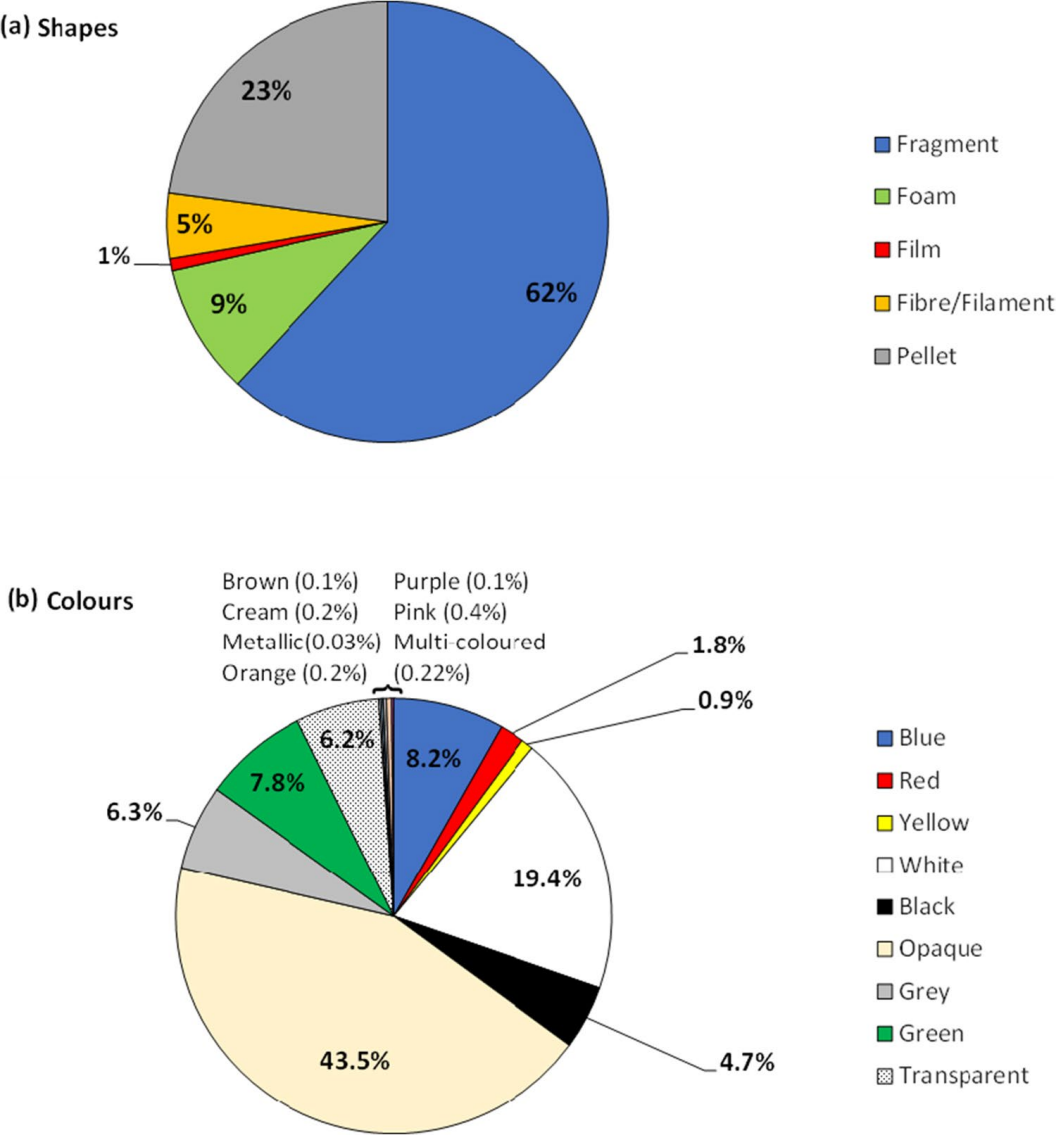
**a** Relative abundances of the morphologies of mp/MePs sampled from the Chessel Bay intertidal shoreline. **b** Colours of the mp/MeP particles enumerated in this study

Across much of the intertidal, microplastic nurdles or pellets were most apparent to the naked eye, leading the observer to assume their numerical dominance compared to other morphologies and furthermore secondary microplastics. Of all 7742 microplastic particles enumerated in both surface and subsurface samples, 77% were of secondary origin (Fig. 4a), where pellets/nurdles accounted for 23%, and plastic degradation reflected in the abundance of fragments (62%), foams (9%), fibres (5%) and films (1%). Additionally, the shoreline microplastics were distinguished across 16 colours and shades (Fig. 4b), where most particles were opaque (43.5%) and white (19.4%), with blue (8.2%), green (7.8%), grey (6.3%), transparent (6.2%) and black (4.7%) in smaller quantities. Yellow, pink, multi-coloured, cream, orange, purple, brown and metallic particles were found in smaller quantities.

#### Microplastic size fractions

**Fig. 5:**
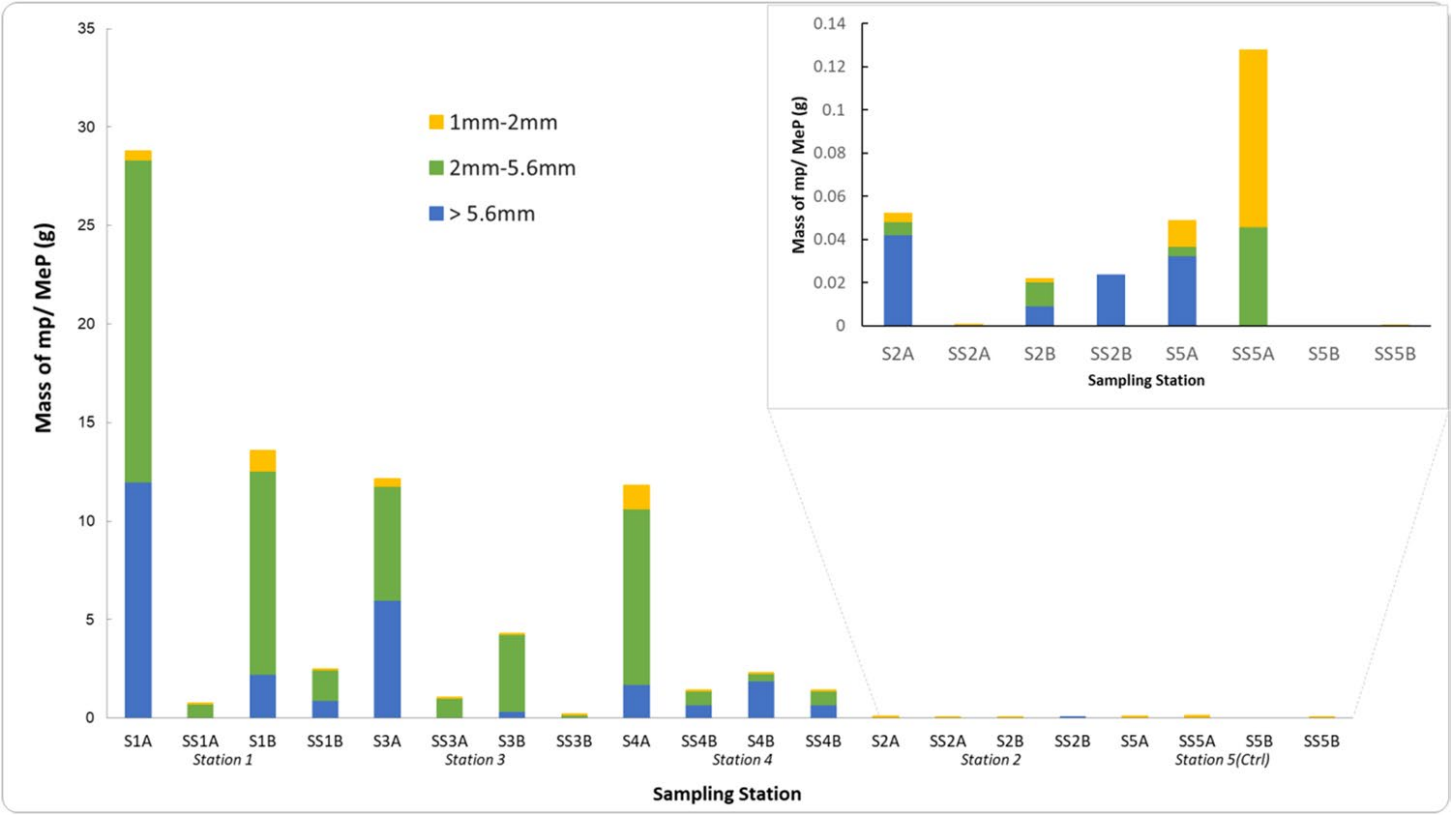
Relative size fractions (by sieve size) of microplastics/mesoplastics in the surface and sub-surface sediment samples of the 5 sampling stations. S1A symbolises the duplicate A of the surface sample at station 1; SS1A symbolises the duplicate sub-surface sample A at station 1, etc. Stations were organised on the *x*-axis in the order of most polluted to least polluted based on initial field observations

Within both the surface and sub-surface samples (Fig. 5), the largest amounts of microplastics by mass were found to be between 2 and 5.6 mm in size, and to a lesser extent those between 1 and 2 mm. The > 5.6 mm, though by definition, not considered as microplastics, were found to be the second-largest size fraction (by mass) of synthetic material. Across the three hotspots, plastics > 5.6 mm were also found in the sub-surface horizons, but those microplastic particles between 2 and 5.6 mm were still most prevalent.

### Organic and inorganic contaminants: PAHs and trace metals

**Fig. 6:**
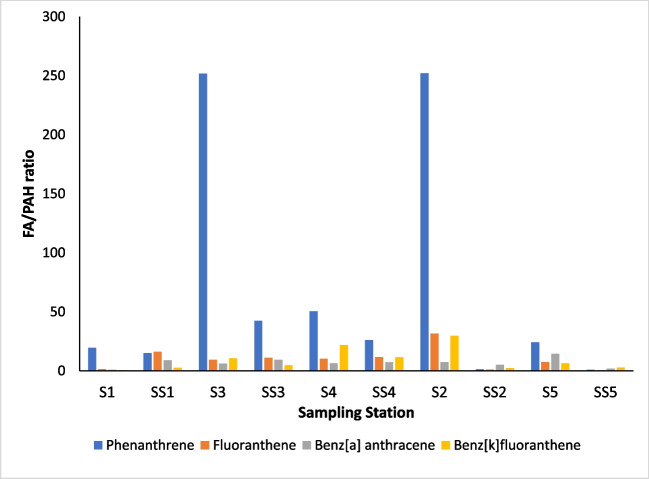
Fatty acid/PAH ratios for phenanthrene, fluoranthene, benz[a]anthracene and benzo[k]fluoranthene across the study area. S1 refers to the surface sediment horizon, while SS1 refers to the sub-surface horizon, and so on

The relative abundances of the four PAHs chosen were quite variable, but in general, the target PAHs were strongly present at each sampling station. Lower FA/PAH diagnostic ratios were indicative of stronger retention of the synthetic PAHs identified, with greater proportions of synthetic PAH signatures generally evident in the sub-surface relative to surface sediments (Fig. 6; Table 4). This was particularly distinctive at the control station, station 5, which recorded the strongest signals of the four PAHs in the sub-surface horizon, indicated by the lowest FA/PAH ratios of the 5 stations. Station 2, which was the least polluted by microplastics, also had strong PAH retention in the sub-surface horizon. The affinity of the microplastic-/mesoplastic-sediment matrix for phenanthrene was the most variable of the PAHs identified. At stations 2 (FA/PAH = 251.99), the least polluted station, and 3 (FA/PAH = 251.79), a microplastic hotspot, the phenanthrene signal of the surface sediment was much more suppressed by the natural palmitic acid than for other hotspots (stations 1 and 4) which had strong phenanthrene signals, as well as the control station (station 5). The signals of the other PAHs, benzo [k] fluoranthene, fluoranthene and benz [a] anthracene, however, were, generally, more prominent across the stations than phenanthrene, and their relative adsorption between the surface and sub-surface sediments quite variable across stations.

#### Microplastic adsorption of trace metals

Trace metal adsorption of the shoreline microplastics was evident well above detectable limits across the sediments of the three microplastic hotspots. The microplastic load from these samples elicited quite variable concentrations across the hotspots and their sediment horizons (Table 1). Al was highest in concentration with 27.7–68.3 ppm in surface microplastics and 24.5–32.4 ppm in sub-surface microplastics. Pb signals were also strong between 2.80 and 11.2 ppm in surface microplastics and 1.21–1.89 ppm on sub-surface microplastics. Moreover, Pb concentrations exceeded the LC_50_ of a similar shoreline gammarid, *Orchomonella pinguis* (Bach et al. 2014). Other elements (Cd, Co, Cr, Cu, Ni) were found adsorbed to microplastics in trace levels compared to other analytes (Table 1). Zn concentrations were 0.260–0.506 ppm (surface) and 0.200–0.344 ppm (sub-surface) across the hotspots.

**Table 1:** Concentrations of trace metals (ppm) retained by microplastics/mesoplastics and the sediments in the surface and subsurface horizons of the Chessel Bay intertidal

Metal	UK Cefas limits (ppm)*	Station 1				Station 3				Station 4				LC_50_ *Orchomenella pinguis* (Bach et al. 2014)	
		Surface		Subsurface		Surface		Subsurface		Surface		Subsurface			
	AL1^**+**^	mp/MeP	Sed	mp/MeP	Sed	mp/MeP	Sed	mp/MeP	Sed	mp/MeP	Sed	mp/MeP	Sed	µmol/L	ppm
Al	-	39.4	2907	24.5	132	27.7	4119	32.4	164	68.3	353	27.4	186	-	-
Cd	0.4	0.009	**1.48**	0.003	0.05	0.008	**1.50**	0.003	0.03	0.011	0.10	0.005	0.24	1.01	0.11
Co	-	0.001	6.30	0.001	0.14	0.002	7.16	0.001	0.13	0.004	0.76	0.001	0.22	-	-
Cr	40	0.003	19.14	0.001	0.45	0.001	20.97	0.002	1.23	0.002	2.59	0.002	0.60	-	-
Cu	40	0.092	**149.0**	0.069	6.37	0.088	**203**	0.108	3.41	0.128	8.95	0.074	3.63	3.41	0.22
Ni	20	0.003	2.40	0.002	1.57	0.002	1.51	0.002	3.27	0.004	2.39	0.003	2.22	-	-
Pb	50	2.797	8.20	1.211	13.1	11.2	5.26	1.894	9.15	4.779	6.85	1.397	8.65	11.1	2.30
Zn	130	0.506	**140.3**	0.234	100.9	0.26	104.45	0.344	111.3	0.41	116.2	0.2	94.05	6.1	0.40

Concentrations in **bold** indicate metal concentrations from the sediment samples of Chessel Bay that exceeded the Cefas guidelines [These are the only guidelines produced in the UK for sediments, and primarily support the disposal considerations for contaminated dredged benthic sediment, so may not necessarily be indicative of potential toxicity to shoreline biota]. Lethal Concentration (LC_50_) values are supplied for a similar supralittoral amphipod *Orchomenella pinguis*

^*^UK Cefas guidelines (MMO 2015)

^**+**^AL1 refers to the action level 1, below which contaminant levels in sediments are of no concern, mp/MeP stands for microplastics/mesoplastics and Sed stands for the sediment residue removed from the surface of the mp/MePs (organic and inorganic material)

Despite the variability in metal concentrations across stations, analysis indicated that these were not significantly different (surface horizon: Kruskal–Wallis test *H*(2) = 0.385, *p* = 0.825; subsurface horizon: Kruskal–Wallis test *H*(2) = 0.213, *p* = 0.899) as the context arises that the shoreline is impacted similarly by the physicochemical conditions from the riverine tides.

Differences in concentrations of all metals analysed between the two sediment horizons were also not statistically significant (Mann–Whitney, *U* = 329, *p* = 0.396).

### Amphipod biometrics and population dynamics

A total of 1258 *O. gammarellus* individuals were sampled from the Chessel Bay supralittoral zone. There was evidence of an active reproductive period as 511 juveniles were enumerated, with 78 ovigerous females and 10 females carrying juveniles in their brood pouch, and 197 not gravid. Two hundred thirty male amphipods were enumerated across the five sampling stations, with station 1, the most polluted station recording the greatest number of individuals, likewise with female amphipods (Table 2). Stations 3 and 4 were less populous than the latter and the areas with the least microplastic pollution (control—station 5 and least polluted—station 2) recorded the lowest numbers.

**Table 2:** Population of *O. gammarellus* with total number of males, ovigerous, non-ovigerous females, juvenile-carrying females and independent juveniles sampled from the intertidal zone of Chessel Bay Nature Reserve. (A and B at each sampling station represent two sample pots set up in duplicate)

Station	Males	Females				Juveniles	Total no. of Amphipods
	*Total*	*Total*	*With eggs*	*With juveniles*	*Neither*	*Total*	
1A	24	33	16	1	16	37	94
1B	126	113	48	6	59	71	310
2A	3	5	1	1	3	22	30
2B	6	5	1	0	4	7	18
3A	14	38	2	1	35	81	133
3B	3	5	0	0	5	48	56
4A	16	14	2	1	11	201	231
4B	18	43	4	0	39	92	153
5A	9	20	3	0	17	70	99
5B	11	9	1	0	8	114	134
Grand total	230	285	78	10	197	743	1258

**Fig. 7:**
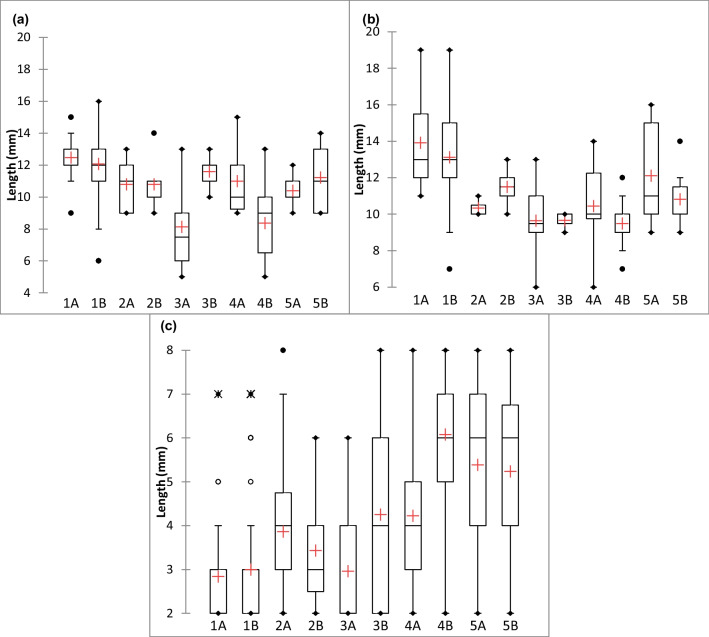
Total body lengths (mm) of **a** female, **b** male and **c** juvenile *O. gammarellus* sampled across the intertidal at Chessel Bay Nature Reserve. The *x*-axis indicates the station number and the duplicate sampling pot at each station

Amphipods were in various growth stages (Fig. 7) of their life cycle, as male lengths were between 6 and 19 mm. Those at smaller lengths that would normally be considered as juveniles had well-defined sexual characteristics but had smaller bodies. Females also varied in length, with observations of smaller bodies (5 mm) and a maximum length of 16 mm. For juvenile amphipods, many specimens were at lengths ~ 2 mm, implying their recent release from the brood pouch of a mature female. On exploring possible associations with microplastic concentrations and the growth (based on length) of the amphipods, there was no statistical significance for the very low positive correlation with the growth of males (*r*(8) = 0.151, *p* = 0.678) and females (*r*(8) = 0.143, *p* = 0.693). Despite some indication that the higher concentrations of microplastics in the sediments resulted in shorter body lengths for juveniles, a Spearman correlation found no statistical significance for this (*r*(8) =  − 0.305, *p* = 0.392).

#### Populations and exposure to organic and inorganic contaminants

Several observations were made based on the population of amphipods sampled for the study with respect to metal concentrations in mp/MePs from the same locations—but demonstrating statistically significant associations was not possible so no firm inferences can be drawn. However, shoreline *O. gamarellus* populations showed some positive correlation with microplastics as a physical contaminant (Spearman correlation, *r*(4) = 0.665, *p* = 0.036). These amphipod populations followed a low distribution in regions of high Pb concentrations (Spearman correlation, *r*(4) =  − 0.478, *p* = 0.338), but population survival seemed to favour locations with higher concentrations of Al (Spearman correlation, *r*(4) = 0.598, *p* = 0.210), Cd (Spearman correlation, *r*(4) = 0.598, *p* = 0.210), Cr (Spearman correlation, *r*(4) = 0.621, *p* = 0.188), Ni (Spearman correlation, *r*(4) = 0.598, *p* = 0.210) and Zn (Spearman correlation, *r*(4) = 0.478, *p* = 0.338).

In relation to PAH signatures in the microplastic-sediment matrix across the five sampling stations, Spearman correlation test indicated low relationships with population abundance, again without statistical significance. There was a low negative correlation between higher populations of amphipods and higher PAH signatures (indicated by low FA/PAH ratios): phenanthrene (*r*(8) =  − 0.419, *p* = 0.229), fluoranthene (*r*(8) =  − 0.369, *p* = 0.294), benz[k]fluoranthene (*r*(8) =  − 0.074, *p* = 0.839) and benz[a]anthracene (*r*(8) = 0.049, *p* = 0.893) all having quite variable relationships. These correlation values may suggest that areas of higher PAH abundance were avoided by the amphipods.

## Discussion

The findings of this study highlight the very high but variable level of mp/MeP contamination in the sediments of the estuarine shoreline of Chessel Bay Nature Reserve and further exemplifies the environmental consequence of the mismanagement of plastic material along the plastic manufacturing and usage producer–consumer stream. Concentrations of mp/MePs determined across the sampling stations validated the initial observations made in situ, for which the three hotspots contaminated with plastic pellets/nurdles had the highest quantities of mp/MePs from primary and secondary sources (stations 1, 3 and 4). Additionally, the highest mp/MeP concentrations were in the surface sediments of these stations, and to a lesser extent, in the sub-surface horizon. Spatial variability of plastic load was especially evident between the least polluted location (station 5), the control station (station 2) and the most polluted station (1) which were near each other (Fig. 1). This may have been due to the filtration effect by vegetation (Stead et al. 2020; Lloret et al. 2021), of which a network of tree roots and shoreline plants shielded the influx of microplastics and lessened the sediment load.

Otherwise, variability in mp/MeP concentration may be consequent to the dynamic nature of estuaries, with the influence of tidal fluctuations, water circulation patterns, infauna burrowing activity, shoreline topography, pollution events and frequencies that may regulate the resuspension, dispersion and settling of plastics (Krelling et al. 2017; Ogbuagu et al. 2022). Ogbuagu et al. (2022) further observed key points for consideration in this discourse: in the saltmarsh-mudflat system of Hythe in Southampton Water, the high retention efficiency of nurdle microplastics (denoted with the pellet morphology in this study) was attributed to the saltmarsh vegetation modulating flow velocity and enhancing sediment accretion, which would in turn limit resuspension of microplastics. Sedimentation rates of organic and anthropogenic material originating from upstream sources, in conjunction with the flood-ebb cycles of the monthly dual spring-neap tides of the study area, may influence the movements of the sediments and result in microplastic accretion over time (NFF 2014; Díaz-Jaramillo et al. 2021).

Within the sediment profile, the differences in microplastic and mesoplastic concentration between surface and subsurface horizons across the stations were variable, thus reflecting the heterogeneity of the shoreline. Surface horizons, though shallower than the sub-surface, retained much higher proportions of loose microplastics and mesoplastics than below, and this may suggest the occurrence of recent pollution events. Microplastics and mesoplastics retained in the sub-surface horizons up to 8 cm had retention influences from several probable trapping variables: shoreline vegetation comprising mostly *S. maritimus*, *P. australis*, *J. gerardii*, *A. pungens* and *A. hastata* influence the sediment accretion (and ultimately, the accumulation of microplastics within) along the Chessel Bay intertidal zone; burrowing and foraging activity by fauna such as the crab *Carcinus maenas* (Ogbuagu et al. 2022) and birds like the oystercatcher (*Haematopus ostralegus*) and the turnstone (*Arenaria interpres*) may further distribute the microplastics deeper into the sediment profile. Lourenço et al. (2017) acknowledged the redeposition of microplastics by shorebird faeces to tidal flats of estuarine regions in Portugal, Mauritania and Guinea-Bissau. Future studies evaluating microplastic deposition and retention in the Chessel Bay Nature Reserve may consider the influences of the shoreline fauna that reside along the site to sediment microplastics concentrations.

Fragments were the most abundant mp/MeP morphology retrieved from the sediments (62%) and combined with foams, films and fibres/filaments; secondary mp/MePs accounted for 77% of the plastic material processed across the five stations (Fig. 4a), contrary to the pre-production pellets/nurdles which are more obvious at first glance along the intertidal but only accounted for 23% of the plastics. This is indicative that fragmentation whether enroute or on-site, due to various degradation processes (photolytic, mechanical, microbial), is the primary contributor (numerically) to plastic pollution along the estuarine shoreline of Chessel Bay. The occurrence of very large, fragmenting pieces of expanded polystyrene floats from discarded or lost buoys and pontoons, are also a source of microplastics by fragmenting into foam pellets which comprised 9% of the mp/MePs in our samples. The plastic signature may have been influenced by surface water run-off from the surrounding Southampton urban area entering one part of the study area, agricultural run-off, littering or fly-tipping upstream and at the reserve (which was evident), and from maritime activity nearby (Fig. 1) (Gallagher et al. 2016). This microplastic generation mechanism reflects similarly to the Hunter Estuary in East Australia, which has a comparable influence of anthropogenic activity and was dominantly impacted by fragments (Hitchcock and Mitrovic 2019).

Future mitigation efforts targeting the plastic pollution at the source are complex due to the multitude of origins of secondary microplastics. The pre-production pellets/nurdles, which accounted for 23% of the microplastics load, mostly indicate losses from nearby plastic manufacturing activities (Gallagher et al. 2016), but some could be washed in on incoming tides from elsewhere. Nevertheless, with an estimated 5–53 billion pellets lost to the UK environment (FIDRA n.d.), environmental regulatory authorities should mandate manufacturers to implement strategies such as that of British Standards Institute PAS 510 standard that minimise pellet loss to the environment (BSI 2021). Reducing the secondary microplastic load will require a synergy of measures across the take-make-waste plastic stream, and locally stakeholder engagement has begun steps to deliver this.

The SCS system enabled sorting into 16 colour sub-groups, and opaque particles were the largest sub-group (43.5%), but white particles were the most dominant of the “coloured” items (19.4%). The latter includes foam microplastics from fragmented maritime floats (see above). Some opaque particles may have originated from the fragmentation of plastic bottles which were evident on the site.

Overall, polymeric material accounted for up to 42% of the sediment matrix of the Chessel Bay intertidal. Size fractionation revealed that particle sizes between 2 and 5 mm had the greatest mass concentrations of the three evaluated size classes. Plastic material > 5.6 mm was second-most abundant by mass and those 1–2 mm, least abundant. However, based on a tally of particles within each size class, microplastics between 1 and 2 mm were most abundant, ranging between 0 and 1456 particles in each sample analysed. Microplastics/mesoplastics between 2 and 5.6 mm were 0–739 particles in abundance. On a quantitative basis, these results follow an inverse relationship that dictates higher abundances of microplastics at smaller sizes (Browne et al. 2010; Hitchcock and Mitrovic 2019). Numerical abundance is particularly relevant in assessing encounter rates between smaller marine organisms and microplastics within the sediments, but does not negate them from their ability to consume larger plastics. Hodgson et al. (2018) showed the ability of *O. gammarellus* to shred 1-cm^2^ pieces of polyethylene, degradable and biodegradable plastic carrier bags. We did not report abundances below 1 mm due to the limitations to accurately qualify microplastics at these sizes with human vision. This will have led to an underestimation of smaller microplastics and nanoplastics, which perpetuates the concern of just how much more bioavailable plastic material may be to macroscopic and microscopic organisms. Nevertheless, the report of larger microplastics, between 1 and 5 mm, as defined by Crawford and Quinn (2017), plus a portion of smaller mesoplastics up to 5.6 mm, serve as immediate indicators of the potentially abundant smaller microplastics (< 1 mm). More importantly, this difference in reporting metric substantiates the continued appeal for standardised microplastic pollution analysis and reporting protocols, not only for study inter-comparisons but to sufficiently inform the degree of environmental impact (Rochman and Boxall 2014).

It is indisputable that spectroscopic elucidation of synthetic polymer chemistry remains an essential complement to isolating suspected microplastics from environmental matrices (Ivar do Sul 2021), which was unfortunately absent from this study given the extraordinary circumstances. Whilst the appeal for method standardisation continues to develop the science of environmental microplastic research, visual classification protocols such as Nor and Obbard (2014) and Lusher et al. (2020) remain valuable characterisation methods for researchers in cash-strapped institutions restricted to these due to the cost of spectroscopic instrumentation or the feasibility of third-party analysis. Where the merit of visual characterisation, however, decreases significantly at smaller particle sizes, researchers should carefully design studies acknowledging this important limitation.

### Microplastics and sorption of contaminants

#### Polycyclic aromatic hydrocarbons

The calculated FA/PAH ratios for phenanthrene, fluoranthene, benz[a]anthracene and benzo[k]fluoranthene highlighted the varying degrees of adsorption by PAHs in the sediment-microplastic matrix. High molecular weight PAHs such as fluoranthene, benzo[k]fluoranthene and benza] anthracene, which are more lipophilic and less labile than phenanthrene, a low molecular weight PAH, were greatly retained in the sediments overall (Ambade et al. 2022). These results are also congruent with Fred-Ahmadu et al. (2022), who found a similar trend in lagoon and beach sediments in the vicinity of Lagos Lagoon and beaches of the Gulf of Guinea coastline. The particularly dominant PAH signal expressed in the sub-surface sediment horizons versus the surface horizons of the five stations could be possibly attributed to sedimentary adsorption and less so, adsorption to microplastics. In the case of station 5, which had the lowest microplastic concentration across the study area, PAH signatures were the greatest of all stations in the surface and sub-surface. In examining this trend, the sorption kinetics of PAHs on microplastics, which was more concentrated in the surface horizons, differs from that of sorption mechanisms with the intertidal substrate. Total organic carbon is considered to have strong positive correlation with PAHs in sediments, and the mechanism for this interaction is still being investigated (Crnkovic et al. 2019). With microplastics, especially in the dynamic nature of an estuary, residence time of microplastics and their sorption capacity, which is highly dependent on the polymer type (Lee et al. 2014), may influence how PAH interacts with the plastic material. Long-term monitoring of the Chessel Bay shoreline can enable capture of potential future microplastic pollution events, allowing for the identification of new plastic material and quantitative analysis of PAHs in sediments and microplastics could inform the relative contributions of beached microplastics to contaminant retention.

Overall, the FA/PAH ratios were indicative of anthropogenic enrichment due a greatly suppressed organic matter signal. Whilst wildfires have been historically uncommon in the UK, diagenesis of organic matter may have been primarily responsible for the background PAH signal. Enhanced concentrations along the Chessel Bay may be due to the tidal transport of pollutants from Fawley oil refinery just south within Southampton Water—which has a history of spillages (George 1971). These signals may also be a historical record of the fires that occurred from the bombing of Southampton in 1940 during World War 2 (Ordnance Survey n.d.) and long-term history of heavy industry locally. Additionally, this enrichment may be due to lubricant and fuel leakages from the nearby marinas and harbours. Attributing specific origins of these PAHs, however, is complex given their numerous sources, and moreover, the chemical and biochemical transformations that they undergo in the marine environment (Soclo et al. 2000; Balmer et al. 2019). Future studies, however, may benefit from the determination of source diagnostic ratios, which are indicative of pyrolytic (incomplete fossil fuel combustion) or petrogenic (crude oil leakage) sources, based on the relative concentrations of alkyl-substituted to unsubstituted PAHs in the sediment (Tang et al. 2018; Balmer et al. 2019). PAH adsorption along the shoreline was mainly attributed to the organic and mineral content of sediment, as microplastics constituted only up to a maximum proportion of 42% of sediment across the stations. Sorption mechanisms by organic matter favour long-term PAH retention described by Yang and Zheng (2010), through the hole-filling domain, where charged sites of the organic matter retard the diffusion process, and also through the partition domain, involving a faster adsorption–desorption rate. Frias et al. (2010) reported that aged and black pellets recovered from beach sediments had adsorbed the highest concentrations of phenanthrene and fluoranthene.

#### Trace metal species

Sediments and microplastics sampled from Chessel Bay were also found to adsorb trace metals, with stronger adsorption in the sediments than on the microplastics. Isotherms often model the adsorption kinetics of metal pollutants between the surrounding medium and particulates, and it remains debatable whether adsorption onto microplastics follows the Langmuir or Freundlich model. Metal cationic species adsorb to microplastics via electrostatic interactions with negatively charged sites (Liu et al. 2021), which may be due to the inherent hydrophobicity of the microplastic particle or from weathering processes (Hee Joo et al. 2021). Hee Joo et al. (2021) highlighted the complexity of the adsorption interface of microplastics, as organic matter may adhere to their surfaces, contributing to additional adsorption site of hydrophobic interaction, pore blockage, electrostatic repulsion and attraction and site competition with anionic species. The residue which was removed during the Milli-Q water rinse of the microplastics maintained higher concentrations of trace metals than the plastics themselves (Table 1). This, therefore, suggests that microplastics in marine environments are a complex adsorption–desorption medium, and future studies investigating associated secondary contaminant levels should consider these mechanisms when analysing and reporting on the role of microplastics as contaminant vectors. Sediment quality guidelines have often been used to infer the degree of ecological risk related to a host of chemical contaminants (Hubner et al. 2009). In this study, the UK Cefas Action Level 1 guideline was selected as a threshold (the only one available in the UK) to ascribe an ecological risk for more sensitive elements of the marine environment. Al was absent from sediment guidelines as it is characteristic of the natural signal of the inorganic aluminosilicate component of sediments and serves as a reference element (Windom et al. 1989). Despite this inherent truth, the results showed that microplastics are carriers of labile aluminium species (Table 1). Cd, Cu and Zn levels extracted from the sediments far exceeded the limit set (Table 1), which may also relate to local anthropogenic sources documented above, plus a large-scale metal recycling facility nearby.

### Potential impacts of microplastics, metals and PAHs on amphipods

**Fig. 8:**
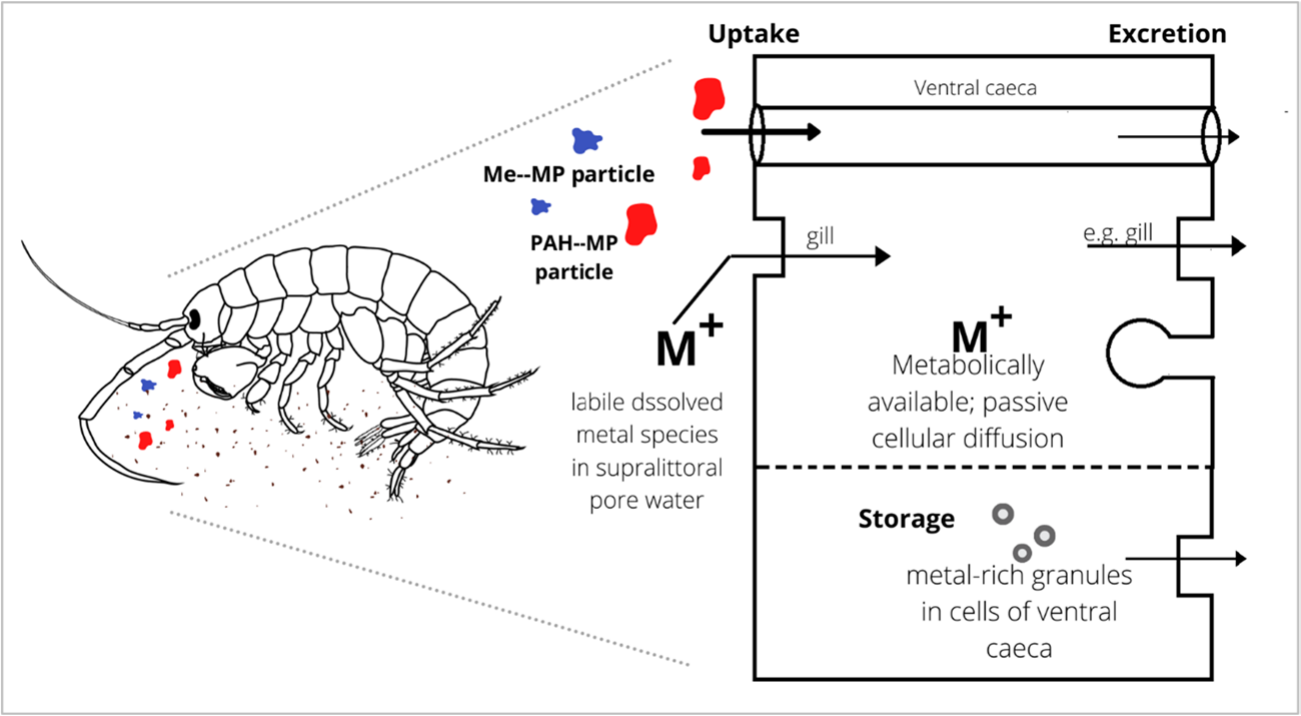
Uptake-excretion model of *O. gammarellus* in relation to ingestion of contaminated microplastics (PAHs; trace metals), in addition to the traditional pathways for metal uptake through diet and diffusion across the gills (Marsden and Rainbow 2004; Rainbow 2018)

**Fig. 9:**
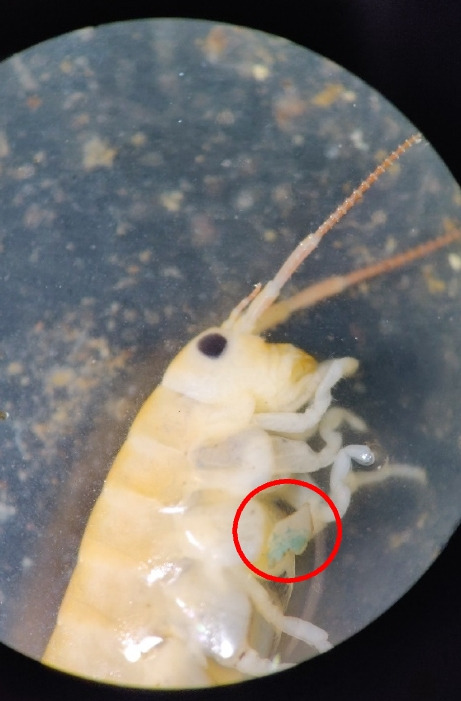
Ovigerous female *O. gammarellus* with microplastic fragment found in brood pouch

Physical and chemical contaminants quantified in this study are potentially detrimental to the survival of *O. gammerellus* at Chessel Bay depending on the capacity of the organisms’ detoxification processes to regulate the rate of entry and excretion or metabolic inactivation (Rainbow et al. 1999). Upon assessing the lethal concentration (LC_50_) values of a similar species of intertidal amphipod *Orchomenella pinguis*, sediment quality guideline limits do not sufficiently qualify the sensitivity of these smaller organisms (Bach et al. 2014). Exposure concentrations from the sediment for Cd, Cu, Pb and Zn, which reflect two essential and two non-essential elements, were all well in excess of the LC_50_. This implies harmful exposure to metal concentrations within the detrital diet of the amphipod, and further assessment of the impact on population dynamics is necessary to quantify long-term effects. Pb surface sediment concentrations at all hotspots exceeded the LC_50_ values of *O. pinguis*, and may relate to the low populations of *O. gammarellus* in this study in sampling locations with high Pb concentrations. Cu concentrations were excessively high relative to these LC_50_ values at station 1 (149 ppm) and station 3 (203 ppm), and still in exceedance, but to a smaller degree, at station 4 (8.95 ppm). Pb signatures may be attributed to pipes that drained the city of Southampton and pre-1980’s municipal run-off, which would have included contaminants from leaded petrol. Microplastics extracted from the three hotspots (stations 1, 3 and 4) similarly reflected adsorption levels above LC_50_ concentrations but to a lower degree (Table 1). Zn concentrations at station 1 and station 4 also exceeded lethal concentration values, with retention at 0.506 ppm and 0.41 ppm respectively. Nevertheless, this does not immediately translate to the bioavailable fraction as sorption–desorption processes and the availability of binding sites within the sediment matrix or neutralising dissolved anionic species in the pore water will influence what is sequestered or labile for adsorption through the organism’s gills in dissolved form, or potentially ingested from the consumption of microplastic particles and detritus (Marsden and Rainbow 2004) (Fig. 8).

Grazing supralittoral amphipods have adapted their physiology to storage of metals essential to their dietary needs, as dissolved metal species suspended in nearby waters are not readily available for uptake through their gills (Weeks and Rainbow 1993). In excess conditions of metal adsorption on organic substrates, this may translate to increased concentrations of metals stored in the caeca of the organism. However, there are no regulatory mechanisms to equilibrate metal concentrations. Weeks and Rainbow (1993) found that caecal copper concentrations in *O. gammarellus* increased significantly in a scenario of highly copper-enriched food. They further observed that this diet caused reduced feeding rate by 1/5 in the amphipods from 5.50 to 1.10 mg g^−1^ h^−1^_._ Given their short life span of 132 days, high ambient metal exposure which translates to unregulated increases in body metal concentrations can retard development and disrupt physiological function. Jelassi et al. (2021) observed significant deformation of cell structure of the hepatopancreas of *O. gammarellus*, with cytoplasm condensation and mitochondria occurring in high concentrations of increased Cd exposure (0.7, 1, 1.3 mg L^−1^ treated soil), and a progressive decrease in some lipid granules in Zn-exposed (300, 400, 500 mg L^−1^ treated soil) individuals. Metal accumulation rates are also variable based on the maturation stage of the amphipod. For juveniles, which moult at higher rates compared to mature adults, their thin bodies and high surface-to-volume ratio may result in greater uptake (Pastorinho et al. 2009). Thus, the under-developed bodies of the juveniles may have lower tolerances and mechanisms to deal with elevated dietary metal exposure. Regardless of the life cycle phase of the amphipods, however, sediment-borne trace metal concentrations were very high and in great excess of LC_50_ concentrations, also posing greater risk to the amphipods than the trace metal adsorbates of the microplastics.

The ecological significance of the quantities of mp/MePs and related adsorbates was evaluated in this study on any apparent effects on populations and biometrics, such as length and physiological changes. There seemed to be some positive correlation between microplastic concentrations and amphipod population levels (Spearman correlation, *R* = 0.665, *p* = 0.036), indicating possible preference by the amphipods for sites with higher levels of plastic contamination. This may be due to improved navigability through the sediment horizon as microplastics facilitate looser material within the rhizosphere for which amphipods may more easily retreat to avoid desiccation and predation. However, the granular nature of mp/MePs along the shoreline may also imply increased permeability, thus better drainage, lower moisture and increased desiccation stress to the amphipods (Carson et al. 2011). At stations 1, 3 and 4, which were considered as the three microplastics hotspots in this study, percent (%) moisture was 31.94%, 37.24% and 37.32% respectively (Table 3). At the least polluted site at station 2 and the control station at station 5, moisture levels were greater (46.38% and 47.36%) respectively. This suggests that uncontaminated shorelines are most beneficial to the survival of these macroinvertebrates, where desiccation stress would be less of a factor. This was similarly reflected in the Spearman’s rank correlation between the moisture levels and the average total (surface and subsurface) sediment microplastic concentration, which indicated strong negative correlation but this was not statistically significant (*r*(3) =  − 0.800, *p* = 0.133). Earlier research by Agnew and Taylor (1986) on a similar cobble stony shore along Great Cumbre Isle observed decreased survival of two gammarid amphipod species, *Echinogammarus pirloti* and *Echinogammarus obtusatus*, in areas of decreased humidity. They further reported a collective response to decreased humidity as individuals clumped together for increased localisation of humidity, and larger groups were observed to have higher survival rate. Attributing behavioural modifications to microplastic concentrations will require a more systemic and detailed assessment of amphipod population dynamics to disqualify or support the synergistic effects of microplastics and their associated adsorbates on the species. Due to a lack of access to FTIR or micro-FTIR spectroscopy in this study, we were unable to progress assessment of gut contents but this could be a valuable area for further research.

### Limitations and recommendations for future work

The researchers acknowledge the impact of the COVID-19 containment measures which limited the accessibility to the desirable spectroscopic validation of mp/MePs sampled from Chessel Bay Nature Reserve. The study took place at a single site and a single season—albeit at a location with a particularly notable set of anthropogenic pressures. Future studies should quantify microplastic concentrations across the gradient and expanse of the Chessel Bay intertidal to capture the spatial variability more accurately and establish quantities and amounts at smaller fractions than were possible here, ideally with some additional characterisation by spectroscopic technique elucidating and confirming a synthetic chemical signature. Furthermore, knowledge of polymer origin would better inform the low affinity of the microplastics for retention of secondary contaminants evaluated in this study. Sediment cores—with additional replicates than were possible here—should also be collected to measure the changes more accurately in microplastic loads along the sediment profile—although this was difficult here due to the shallow depth available, below which were gravels and shingle. Repeated monitoring may also capture the temporal variations associated with recirculation of microplastics alongside modelling the fluxes of microplastics into the estuarine system based on the hydrodynamics of the river, as well as fluctuations in humidity which may affect the survival of invertebrates. Future work may need to consider the response of shoreline amphipods to elevated metal concentrations and closely investigate the influence of microplastics in the burrowing and locomotive activity of these amphipods.

## Conclusions

This study provided a quantitative record of the microplastic contamination across the intertidal zone of the Chessel Bay Nature Reserve, a location which is internationally designated for nature conservation, but badly impacted by human activity. Microplastic concentrations quantified in this study were congruent with initial site observations where high densities of microplastic nurdles or pellets were observed in areas denoted as ‘hotspots’. It also accounts the influence of shoreline vegetation in trapping microplastics within this estuary, where sparsely vegetated areas recorded lower microplastic concentrations. Microplastic concentrations, though abundant across the study area, accounted for less than half of the total sediment matrix across the sampling stations.

Secondary microplastics (fragments, fibres, film, foam) comprised the majority of the total microplastics sampled, thus contradicting the immediate observations of pre-production pellets (or nurdles) across the study area and reflect the mechanisms of weathering, biotic interactions and fragmentation as primary drivers of shoreline microplastic pollution. The range of colours and morphologies highlight the numerous sources from which microplastics may originate, but nurdles nonetheless represented 23% of the polymer material in our samples—and as their sources are relatively easy to identify, represent a potential ‘easy hit’ for regulators in terms of reducing pollution by working with the industry to eliminate accidental spillages.

The retention of secondary contaminants (as trace metals and PAHs in this study) within the shoreline sediments was attributed greatly to their high affinity for the active sites in natural organic and inorganic material rather than the microplastics themselves. They nevertheless are an environmental contaminant of concern for this region. Whilst demonstrating a strong PAH signal in some locations, for certain compounds, results did not substantiate an effect on the development and growth of *O. gammarellus*. Similarly, it remains inconclusive with regards to physiological impacts due to patchily distributed high concentrations of potentially harmful trace metals such as Cd, Cu, Pb and Zn. Despite this, however, microplastic concentrations, in conjunction with the secondary contaminants and high nutrient inputs, are indicative of great anthropogenic pressure along the estuarine shoreline of Chessel Bay Nature Reserve. This study highlights the complex nature of the existence of microplastics in the marine environment and the complexity to which ecotoxicological impacts can be attributed solely due to their retention in these ecosystems.

## Data Availability

The dataset is stored at the University of Southampton Research Data Repository and is made accessible under CC-BY license at 10.5258/SOTON/D2158.

## Appendix 1

See Table 3

**Table 3:** Sampling stations with location, moisture content, depth of sediment horizons, contamination status and vegetation classification

Station	Description of station	Station type	Location		Taxonomic classification of vegetation	Average percent moisture of sediment (%)	Sediment profile region sampled	Depth (cm)
			Easting	Northing				
1	Highly contaminated with macroplastics and microplastics and a dominant portion of nurdles	Hotspot	001.37734	50.91647	*Phragmites australis* (70%); *Scirpus maritimus* (20%); *Juncus geradii* (10%)	31.94	Surface	2–3
							Sub-surface	3–6
2	Little visible microplastic or microplastic contamination; partially sheltered by a tree	Least contaminated area	001.37709	50.91633	Scirpus *maritimus* (75%); *Phragmites australis* (20%); *Juncus gerardii* (5%)	46.38	Surface	0–3
	Gravel below 5 cm						Sub-surface	3–5
3	Mostly macroplastic debris and microplastic nurdles	Hotspot	001.37537	50.91566	Sparse *Phragmites australis* (15%)	37.24	Surface	0–3
							Sub-surface	3–6
4	Mostly sheltered shingle but with highly visible foam debris	Hotspot	001.37706	50.91659	*Phragmites australis* (60%); *Agropyron pungens* (30%) *Atriplex hastata* (10%)	37.32	Surface	0–2
							Sub-surface	2–8
5	On-site control, representing the least impacted site by visible microplastic fragments	Control	001.37700	50.91635	*Scirpus maritimus* (75%); *Phragmites australis* (20%); *Juncus gerardii* (5%)	47.36	Surface	0–1
	Gravel below 5 cm						Sub-surface	1–5

## Appendix 2

See Fig. 9

## Appendix 3

See Table 4

**Table 4:** FA/PAH ratios of microplastic-contaminated surface and subsurface sediment samples at Chessel Bay Nature Reserve

Sample ID	Phenanthrene	Fluoranthene	Benz[a] anthracene	Benz[k]fluoranthene
S1	19.53	1.49	0.75	0.62
SS1	14.84	16.07	8.77	2.40
S2	251.99	31.40	7.24	29.51
SS2	1.29	1.04	5.10	2.34
S3	251.79	9.14	6.01	10.42
SS3	42.27	10.88	9.30	4.53
S4	50.43	10.01	6.24	21.67
SS4	26.03	11.56	7.15	11.35
S5	24.14	7.30	14.40	6.24
SS5	0.68	0.29	1.78	2.77

The FA/PAH term represented here is that of the signature of palmitic acid relative to phenanthrene, fluoranthene, benz[a]anthracene and benzo[k]fluoranthene. S1 refers to the surface horizon of station 1, and SS1 refers to the sub-surface of station 1, and* so on*
